# Abdominal perforation after rupture of a diamond-studded wire: a case report

**DOI:** 10.1186/1757-1626-1-307

**Published:** 2008-11-13

**Authors:** Moritz Schmelzle, Hanno Matthaei, Roy Y Tustas, Marcus Schmitt, Volker Müller-Mattheis, Wolfgang Linhart, Claus F Eisenberger, Wolfram T Knoefel, Jan Schulte am Esch

**Affiliations:** 1 Department of General Surgery, Heinrich-Heine-University Düsseldorf, Germany; 2 Department of Trauma and Hand Surgery, Heinrich-Heine-University Düsseldorf, Germany; 3 Department of Gastroenterology, Hepatology and Infectious Diseases, Heinrich-Heine-University Düsseldorf, Germany; 4 Department of Urology, Heinrich-Heine-University Düsseldorf, Germany

## Abstract

**Introduction:**

There are numerous cases of abdominal injuries due to bullets. Abdominal injuries due to bullets are a diagnostic and therapeutic challenge. Here, an unusual case of an abdominal perforation caused by a metal projectile, lead to confusion in the interpretation of the preoperative computer tomography.

**Case presentation:**

We present an unusual case of a 32-year-old male worker who sustained a "shot" to the left upper abdominal quadrant, as a result of a work-related accident. The projectile derived from a special wire that tore during operation. One chain element happened to accelerate towards the patients belly and perforated the abdominal wall. Computer tomography located the radiopaque projectile to the cortex of the left kidney and showed a lesion of the tail of the pancreas. The presence of intraperitoneal free air suggested a gastrointestinal perforation. Immediate open exploration of the peritoneal cavity and the retroperitoneal space revealed perforating lesions of the anterior and posterior gastric wall, as well as the pancreatic tail. The projectile was finally retrieved in the upper pole of the left kidney. The patient had a good clinical course subsequent to surgery and was discharged in good general condition.

**Conclusion:**

This case represents a rare form of a retained bullet injury and corroborates the need of sufficient measures of worker-protection in area of diamond-studded wire cutting devices.

## Introduction

**Figure 1:**
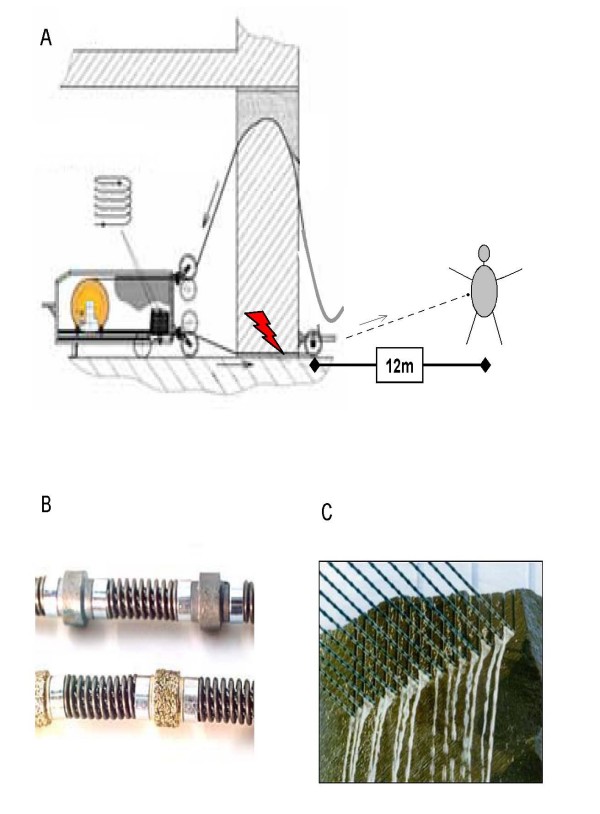
**A-C**. In this case the wire ripped subsequent to extreme tension, so one chain element happened to accelerate towards the patients belly and perforated the abdominal wall Flash indicates the position of wire rupture. The dashed line represents the track of the projectile (A). These special diamond -studded wires are used for cutting large scale rock like material at the wire turn speed of 20–25 m/sec. Up to fourty diamond-studded pearls (5–11 mm in diameter) are assembled (B, C).

There are numerous cases of abdominal injuries due to bullets. Here, an unusual case of an abdominal perforation caused by a metal projectile, lead to confusion in the interpretation of the preoperative computer tomography because of unknown mechanism of injury. We report the case of a male worker hit by a chain link after rupture of a special diamond-studded wire. These kind of wires are used to cut up houses into pieces at a wire turn speed of 20–25 m/sec. Up to fourty diamond-studded pearls (5–11 mm in diameter) are assembled [Figure 1A–C]. Dismantling of pearls during rupture of these wires is not reported yet.

## Case presentation

**Figure 2:**
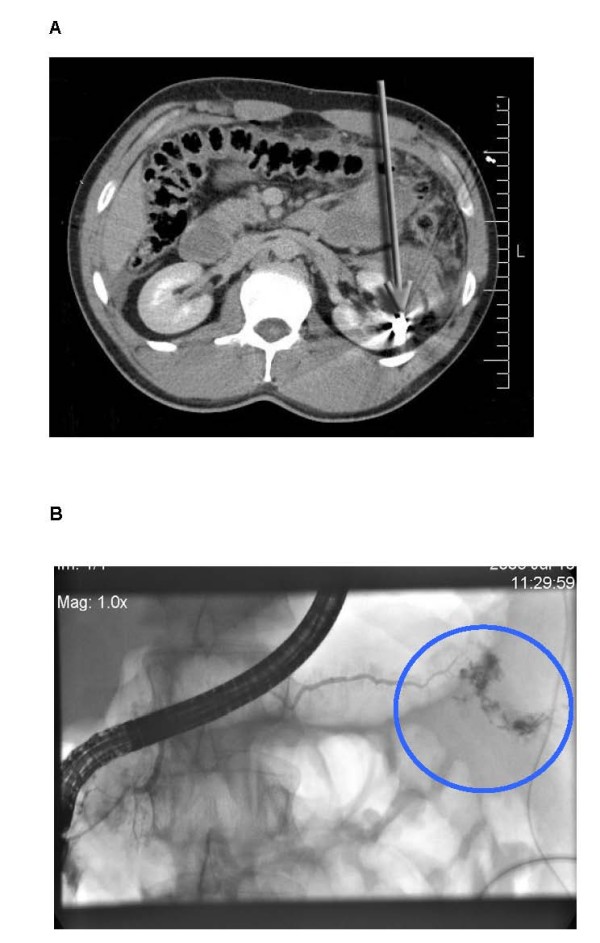
**A**. The CT scan of the abdomen of the 32-year old worker, who was hit by a chain link after rupture of a special diamond-studded wire, is displayed. Free air and fluid are obvious in the peritoneal cavity. A lesion of the pancreatic tail has been suspected. The arrow marks the metal fragment in the upper pole of the left kidney and indicates the track of the projectile. **2B**. A low output pancreatic fistula treated by endoscopic retrograde pancreaticoscopy with placement of a decompressing stent to the common duct four days after surgery.

A 32-year old male patient, working for a building enterprise, broke down on the job. As the patient experienced strong pain and was tachycardic and hypotensive, he was transferred intubated by helicopter to our institution for primary care. On admission the patient was in hemodynamically stabilised condition, though an ambigiuous shot-wound like entry wound in the upper left quadrant of about 5 mm in diameter was mentioned. By computerized tomography an unique metalic fragment in the upper pole of the left kidney with subcapsular bleeding was evidenced [Figure 2A]. Abdominal free air was indicative for a hollow organ perforation. A lesion of the pancreatic tail was suspected. The liver and spleen were also free of signs for injury in the pre-surgical diagnostic procedures. The presence of fluid within the subhepatic space, Morrison's pouch, was suspicious for blood. It was decided to perform an exploratory laparotomy.

Exploration revealed about 400 ml fresh, partly coagulated blood. Perforations of the ventral (entrance wound) and dorsal (exit wound) gastric wall respectively (5 mm in diameter) were identified and closed by single suturetechnique utilising resorbable material. Broad exposure of the bursa omentalis showed a wound correspondending to the dorsal stomach lesion. After mobilisation of the spleen and the pancreatic tail, a tangential injury of the pancreas could be demonstrated. The major pancreatic duct was not lacerated by the metal fragment. The adipose capsule of the kidney was completely blood-filled. We split Gerota's fascia and mobilized the kidney. The upper pole showed an entrance hole on the anterior side without an exit wound. A metal fragment was removed guided by x-ray fluoroscopy. The pancreatic and the renal injuries were treated without sutures, but drained adequately transcutaneously with soft silastic drains. The exocrine pancreas was blocked by subcutaneous application of 100 μg per die octreotide for the following seven days to minimize the development of post-operative pancreas-fistula.

After surgery, the patient was transferred to our intermediate care unit for 11 days. Retrospectively the mechanism of injury was reconstructed. Not involved in the trenching of a dilapidated building with a special wire, the worker was hurt while walking by in a distance of about 12 meters to a diamond wire divice in motion in the moment of wire rupture. A fragment hit the person by maximum speed and penetrated into the abdomen.

The patient further stabilized under antibiotic coverage by Piperacillin and Sulbactam with declining infection signs in clinical chemistry. A low output pancreatic fistula closed 8 weeks after endoscopic retrograde pancreaticoscopy with placement of a decompressing stent to the common duct four days after surgery [Figure 2B]. The patient was transferred to regular ward and discharged from hospital in good condition and closed wounds after closure of the pancreatic fistula. The stent could be removed on an outpatient basis without any complications after 4 month.

## Discussion

Penetrating injuries to the abdomen are a diagnostic and therapeutic challenge. The majority of these injuries require surgical intervention [1]. Penetrating injuries can be classified into stab wounds, gunshot wounds and impalement injuries. Gunshot wounds are the most frequent injuries of these [2]. In the majority of cases they are caused by an act of aggression or in suicide intent [1]. Most frequently, a single organ is affected such as the liver, kidney or spleen [2]. In case of penetration, multi organ injuries still remain a problem, as they considerably increase mortality.

Traumatic injuries of the pancreas are uncommon but serious findings. However, especially pancreatic traumas are often associated with penetrating trauma. There is a high complication rate after such injuries due to pancreatic fistulas, pseudocysts, bleeding and serious infections [3-5]. The importance of draining pancreatic lesions without ductal injury is emphasized, and the severity of the pancreatic lesion needs to be classified for adequate treatment [[6,7] and Table 1: Pancreatic organ injury scale. American Association for the Surgery of Trauma [8]]. Drainage has become widely accepted as the management of choice in pancreatic injuries where there is no suspicion of ductal injury [9,7,10].

**Table 1:** Pancreatic organ injury scale.

Grade	Description
I	Minor contusion or laceration without ductal injury
II	Major contusion without duct injury or tissue loss
III	Distal transection or parenchymal injury with duct injury
IV	Proximal (to right of superior mesenteric vein) transection or parenchymal injury, not involving ampulla
V	Massive disruption of pancreatic head

Pancreatic organ injury scale. American Association for the Surgery of Trauma [10].

## Conclusion

In the present case we demonstrate an unusual mechanism of injury, which has not been previously reported and is a result of an industrial accident. It should be noted that the diamond armed pearls lined up on the wire on intact devices can be accelerated to maximum speed in a whip-like mechanism after a tear of the basic wire. The injury pattern in our patient was comparable to those caused by gun shots. Surgeons should keep in mind the enormous motive force which follows from breaking off of moving parts and leads to the demonstrated degree of damage. Furthermore, the present case highlights the potential "firing range" those diamond-studded wire cutting devices bear and the need of sufficient measures of worker-protection in the area of operation.

## Competing interests

The authors declare that they have no competing interests

## Consent

Written informed consent was obtained from the patient for publication of this case report and accompanying images. A copy of the written consent is available for review by the Editor-in-Chief of this journal.

## Authors' contributions

MS, RYT, VMM, WL and JSAE performed the surgery, conducted the acquisition, analysis and interpretation of data and drafted the manuscript. MAS performed the stent placement by endoscopic retrograde pancreaticoscopy. HM, CFE and WTK made substantial contributions to the conception, acquisition and interpretation of data and revised the manuscript critically. All authors read and approved the final manuscript.
